# Development and use of a content search strategy for retrieving studies on patients' views and preferences

**DOI:** 10.1186/s12955-017-0698-5

**Published:** 2017-08-30

**Authors:** Anna Selva, Ivan Solà, Yuan Zhang, Hector Pardo-Hernandez, R. Brian Haynes, Laura Martínez García, Tamara Navarro, Holger Schünemann, Pablo Alonso-Coello

**Affiliations:** 1 Clinical Epidemiology and Cancer Screening, Corporació Sanitària Parc Taulí, Parc Taulí, 1, Edifici Santa Fe, planta baixa. 08208 Sabadell, Barcelona, Spain; 2 Iberoamerican Cochrane Centre, Biomedical Research Institute Sant Pau (IIB Sant Pau), Barcelona, Spain; 3 Research Network on Health Services in Chronic Diseases (REDISSEC), Barcelona, Spain; 4 CIBER Epidemiología y Salud Pública, (CIBERESP), Barcelona, Spain; 5 Department of Clinical Epidemiology & Biostatistics, McMaster University, Hamilton, ON Canada; 6 Health Information Research Unit, Department of Clinical Epidemiology & Biostatistics, McMaster University, Hamilton, ON Canada

**Keywords:** Patient preference, Decision-making, Information storage and retrieval, Search engine, Terminology as topic

## Abstract

**Background:**

Identifying scientific literature addressing patients’ views and preferences is complex due to the wide range of studies that can be informative and the poor indexing of this evidence. Given the lack of guidance we developed a search strategy to retrieve this type of evidence.

**Methods:**

We assembled an initial list of terms from several sources, including the revision of the terms and indexing of topic-related studies and, methods research literature, and other relevant projects and systematic reviews. We used the relative recall approach, evaluating the capacity of the designed search strategy for retrieving studies included in relevant systematic reviews for the topic. We implemented in practice the final version of the search strategy for conducting systematic reviews and guidelines, and calculated search’s precision and the number of references needed to read (NNR).

**Results:**

We assembled an initial version of the search strategy, which had a relative recall of 87.4% (yield of 132/out of 151 studies). We then added some additional terms from the studies not initially identified, and re-tested this improved version against the studies included in a new set of systematic reviews, reaching a relative recall of 85.8% (151/out of 176 studies, 95% CI 79.9 to 90.2). This final version of the strategy includes two sets of terms related with two domains: “Patient Preferences and Decision Making” and “Health State Utilities Values”. When we used the search strategy for the development of systematic reviews and clinical guidelines we obtained low precision values (ranging from 2% to 5%), and the NNR from 20 to 50.

**Conclusions:**

This search strategy fills an important research gap in this field. It will help systematic reviewers, clinical guideline developers, and policy-makers to retrieve published research on patients’ views and preferences. In turn, this will facilitate the inclusion of this critical aspect when formulating heath care decisions, including recommendations.

**Electronic supplementary material:**

The online version of this article (doi:10.1186/s12955-017-0698-5) contains supplementary material, which is available to authorized users.

## Background

Health care decision-making is complex and involves considering several criteria simultaneously. Recently, the GRADE working group has published a series of manuscripts about the development of frameworks to structure and make more explicit the process of moving from evidence to decisions (including recommendations) [1–3]. Among the suggested criteria to take into consideration when adopting these frameworks, two are particularly relevant to patients. One is the relative importance that patients (or those affected by the decision) place on the main outcomes, crucial when balancing desirable and undesirable effects [4, 5]. The other is whether patients (or other stakeholders) find acceptable the intervention (or the alternative considered) [6, 2]. The GRADE working group used the concept "values and preferences" and conceptualized it as “*the processes that individuals use in considering the potential benefits, harms, costs, limitations, and inconvenience of the management options in relation to one another*” [7], is closely related with both the relative importance of outcomes importance and acceptability and can be considered an umbrella term.

The consideration of how much patients or those affected by a decision value outcomes is an often-ignored aspect when healthcare panels formulate recommendations (or other type of decisions) [8–11]. In particular, healthcare guidelines fail to properly incorporate stakeholders’ views [12–16]. One of the potential reasons for this poor uptake is likely to be the difficulty in identifying relevant scientific literature, due to the wide range of potentially relevant study designs [17], and their poor indexing [18].

There are different ways of capturing peoples’ views about the importance of outcomes (Table 1). Outcomes can be operationalised as health state utility values, which can be obtained using direct or indirect techniques. The former include the standard gamble [19], the time trade-off [20], or visual analogue scales [21], among others. The latter include multi-attribute instruments in which the relative importance is obtained by regression analysis from quality of life questionnaires, such as the EQ-5D (EuroQol), the SF-6 health survey, or the Health Utility Index (HUI-2 and HUI-3) [22]. People’s views on outcome importance can also be elicited using non-utility quantitative measures, typically provided by surveys or questionnaires [23]. Other non-utility measures include direct choice methods [24] such as those used in decision aids [25], which can provide information about the relative disutility of outcomes. Lastly, qualitative research can provide information on preferences, opinions, perceptions, and attitudes [26].

**Table 1:** Measures capturing people’s views about healthcare outcomes

Utility measures		Non-utility, quantitative measures	Qualitative findings	
Direct techniques	Indirect techniques	Direct choice techniques	Interviews	Discussion groups
Standard gamble, time trade off, or visual analogue scales.	Pre-scored multi-attribute instruments [EQ-5D (euroQoL), the SF-6 health survey, or the health utility index (HUI-2 and HUI-3)]	Decision aids and direct choice studies. Surveys or questionnaires.	Structured, semi-structured, unstructured, or in-depth.	Focus groups.

Given the wide range of study designs, it is not surprising that standard strategies for systematically eliciting scientific literature on views and preferences are not yet available. Despite the availability of some search filters, these do not cover all the relevant aspects that need to be considered. Some fall short in capturing the methodologies available to obtain the different possible measures [27, 28], while others focus specifically on one type of measure [29, 30].

Systematically identifying evidence addressing views and preferences through standardised approaches is essential because it would facilitate the conduct of systematic reviews while enhancing its efficiency, structure and transparency and would allow guideline panels to develop evidence-based recommendations [5, 31]. We therefore developed a content search strategy to systematically identify this type of evidence in PubMed.

## Methods

### Development of the initial search strategy

Using an iterative process we developed an initial list of terms, either controlled vocabulary (MeSH terms) and text words. We first explored how related research on this topic was indexed in Pubmed [32–39] and checked search strategies of relevant systematic reviews [31, 40–44]. We also inspected previous search strategies or filters [27–29] as well as other searches used in other related projects and experiences [45–47].

Three authors (AS, IS, and PAC) assembled the initial list of all the terms. This list was refined through discussion among all authors, some of whom have extensive experience in healthcare guideline development and methodology.

### Testing the performance of the search strategy

We assessed the search strategy performance measuring its relative recall, which allows evaluating and refining the search filters performance against the set of studies eligible for inclusion in relevant systematic reviews [48–52]. This approach considers that the methods implemented to identify studies in a systematic review are an efficient alternative to the traditional gold standard obtained from handsearching suggested by other authors [49]. Then, the articles identified in multiple information sources in systematic reviews are representative of the available evidence about a topic, and then the included studies can be used as the reference set to assess the performance of a new search strategy. Relative recall is the proportion of articles that a specific search retrieves of the total relevant studies identified by a systematic review [50]. (Table 2).

**Table 2:** Relative recall calculation

Relative recall = Number studies included in the SRs retrieved by the search × 100 Number of studies included in the SRs indexed in PubMed	

To assess the relative recall of our search strategy we first selected a convenience sample of six relevant systematic reviews that included different and relevant study designs, methods and measures related to our topic of interest [31, 40–44]. All six reviews had clear inclusion criteria and provided a clear list of included studies. Their scope and methods are summarized in Table 3.

**Table 3:** Characteristics of the reference set of systematic reviews

Systematic reviews	Topic	Techniques for elicitation of patient views and preferences of included studies
McLean 2012 [29]	Antithrombotic therapy	Elicitation of health state utility values; Choices patients made when presented with decision aids; Interviews; Surveys.
Peasgood 2010 [41]	Breast cancer	Elicitation of health state utility values.
Petrillo 2011 [42]	COPD	Elicitation of health state utility values.
Bremmer 2007 [38]	Prostate cancer	Elicitation of health state utility values.
Brooker 2013 [39]	COPD	Choices patients made when presented with decision aids; Questionnaires, surveys; Structured and semi-structured interviews.
Morton 2010 [40]	Chronic kidney disease	Qualitative research: focus groups, interviews

We determined which studies included in the reference set of systematic reviews were indexed in PubMed in order to obtain a valid denominator for calculating the relative recall. We aggregated the PubMed unique identifier of each study (PMID) using the Boolean operator OR, and combined them with the search string using the Boolean operator AND.

We determined how many of the primary studies included in the relevant systematic reviews of interest that were indexed in PubMed were retrieved using our search strategy (Table 2). We expressed recall as a proportion and also calculated its 95% confidence interval. We examined the terms used by the primary studies that were not retrieved by the search strategy, and adapted the search accordingly adding the more pertinent terms. We included the search strategy obtained in Additional file 1.

To avoid biases resulting from the initial selection of the reference systematic reviews used to calculate the relative recall and improve the designed search strategy we re-tested its performance against a set of 10 new systematic reviews, similar to those used in the previous step. We conducted a search in PubMed in order to locate systematic reviews of utility measures and other measures. We randomly selected a set of these reviews according to a sequence generated using Microsoft Excel. For the selected reviews we repeated the process described above to calculate the relative recall from our search strategy.

### Search strategy implementation in practice

The search strategy obtained was implemented in the following knowledge synthesis projects: 1) the development of a systematic review about the relative importance of outcomes in COPD patients; 2) the development of a practice guideline for the ARIA (Allergic Rhinitis and its Impact on Asthma) initiative; and 3) the update of a clinical guideline on pregnancy and postnatal care [53]. Additionally, we used this strategy to identify systematic reviews for the development of a repository of systematic reviews and primary studies on patients and other stakeholders’ views about health care, linked to Epistemonikos.

For each of these projects, titles and abstracts retrieved using this strategy were independently screened by two researchers using a reference-managing software (EndNote). Eligibility was discussed through standard methods according the inclusion criteria for each of the projects described above. For the purposes of this paper we calculated the precision of the search strategy for each project (percentage of relevant articles in the complete set of articles retrieved) and the number of references needed to read to obtain a relevant reference (NNR) [54–56] (Table 4). We calculated these estimates for the complete search string and for each of its domains separately, accounting for the different approaches to capture patients’ views and preferences.

**Table 4:** Precision and Number Needed to Read (NNR)

Precision: (number of relevant studies identified/total number of references retrieved from the search) X 100 Number Needed to Read (NNR): number of references needed to screen to find one relevant study (1/precision).	

## Results

### Search strategy development and performance testing

We assembled an initial version of the search strategy, grouping its terms in two domains corresponding to major issues in the topic (“patient preferences and decision making” and “health state utilities”.

We tested the relative recall of the search initial version against the included studies of an initial set of six systematic reviews. The reviews included 162 studies of which 151 (93%) were indexed in PubMed. This initial version of the search strategy retrieved 132 of the reference studies, resulting in a relative recall of 87.4% (95% CI 81.2% to 91.8%).

After assessing the studies not retrieved by the initial version of the search strategy we added some new terms, all related to the patient preferences and decision-making domain. We include the improved version of the search strategy (Additional file 1) that showed a relative recall of 92% (95% CI 86.4% to 95.4%) (Table 5). We also obtained the relative recall for each of the two domains form the search strategy, with a better performance for the block of terms related to patient preferences and decision-making, compared to the terms related to health state utility values (85.4% versus 44.4%).

**Table 5:** Performance of the search strategy

Systematic review	Topic areas	Studies included (n)	Studies indexed in Pubmed (n)	Studies retrieved by the search strategy n (%)	Patient preferences and decision making domain n (%)	Health state utility values domain n (%)
Initial test of the relative recall						
McLean 2012 [29]	Antithrombotic therapy	50	49 (98%)	48 (97.9)	48 (97.9)	3 (6.1)
Peasgood 2010 [41]	Breast cancer	50	44 (88%)	36 (81.8)	32 (72,73)	34 (77,27)
Morton 2010 [40]	Chronic kidney disease	18	15 (83%)	15 (100.0)	15 (100.0)	1 (6.7)
Petrillo 2011 [42]	COPD	11	11 (100%)	10 (90.9)	5 (45.4)	9 (81.8)
Brooker 2013 [39]	COPD	12	12 (100%)	11 (91.7)	11(91.7)	2 (16.7)
Bremmer 2007 [38]	Prostate cancer	21	20 (95%)	19 (95.0)	18(90.0)	18 (90.0)
Relative recall results RR*(95% CI) (n)		162	151 (93%)	92% (86.4-95.4) (139)	85.4% (78.9-90.1) (129)	44.4% (36.4-52.3) (67)
Re-test						
Shaw 2016 [57]	CV disease and daibetes	14	13 (93%)	11 (86,62)	11 (86,62)	0 (0.0)
Smith 2015 [58]	Phyisical activity in joint replacement	13	13 (100%)	9 (69,23)	9 (69,23)	1 (7,69)
Finlayson 2015 [59]	Metastatic cancer	15	15 (100%)	8 (53,3)	8 (53,3)	0 (0,0)
Köberich 2015 [60]	Patient-centered nursing care	12	12 (100%)	11 (91,6)	11 (91,6)	0 (0,0)
Loewen 2017 [62]	Antithrombotic therapy in atrial fibr.	28	26 (93%)	25 (96,15)	25 (96,15)	11 (42,3)
Cowley 2016 [61]	Esophagogastric cancer	2	2 (100%)	0 (0)	0 (0)	0 (0)
Brown 2017 [63]	Lung cancer	27	27 (100%)	23 (85,15)	11 (40,74)	20(74,07)
Jeong 2016 [73]	Colorectal cancer	57	56 (98%)	53 (94,64)	26 (46,42)	51 (91,07)
Carter 2015 [65]	Gastric cancer	8	7 (87.5%)	7 (100)	3 (42,8)	7 (100)
Ward 2017 [64]	Brain trauma	5	5 (100%)	4 (80)	4 (80)	2 (40)
Relative recall results RR*(95% CI) (n)		181	176 (97%)	85,8% (79.9-90.2) (151)	61.4 (54.0-68.3) (108)	52.3% (44.9-59.5) (92)
Total. Relative recall results RR*(95% CI) (n)		343	327 (95%)	88,7% (84.8-91.7) (290)	72.5% (67.4-77.0) (237)	48.6% (43.2-54.0) (159)

*RR: Relative Recall

We then tested the relative recall of the improved version of the search strategy against the studies included in a new set of 10 systematic reviews that included a total of 181 primary studies [57–65]. The relative recall for this second test was 85,8% (95% CI 79.9% to 90.2%) (Table 5). In this second test the difference in the relative recall between the two domains was lower (61.4% for patient preferences versus 52.3% for health state utility values) (Table 5).

If we consider altogether the studies included in the 16 systematic reviews used as reference at the two steps of the test (*n* = 327), then the relative recall of the search strategy was of 88.7% (95% CI 84.8% to 91.7%), being the domain on patient preferences and decision making the one that retrieves a higher proportion of relevant studies (relative recall of 72.5% (95% CI 67.4% to 77.0).

### Search strategy implementation

We used this search strategy in the development of a systematic review and two clinical guidelines that considered the topic of patients’ views and preferences. For these projects we were not able to obtain a reference standard and calculate the relative recall of our strategy. Instead, we obtained its precision and NNR as valid and relevant performance indicators. The results derived from each search showed a low precision for the strategy (Tables 6, 7 and 8).

**Table 6:** Use of the search in the development of a systematic review on COPD

COPD systematic review^a^	Retrieved references (n)	Relevant references (n)	Relevant studies on non-utility measures	Relevant studies on utiltiy measures	Relevant qualitative research	Precision (%) 95% CI	NNR
Global search strategy	12,574	252	93	66	110	2.0 (1.77-2.26)	49.8
Patient preferences domain	11,789	203	87	23	93	1.52 (1.32-1.76)^b^	65.5^b^
Utility values domain	1008	70	11	59	2	5.85 (4.56-8.08)^c^	17.1^c^

^a^Search period: from inception until 01/01/2015

^b^The precision for the patient preferences domain is calculated as: (number of non-utility studies + qualitative studies)/number of retrieved references by the patient preferences domain. The NNR is calculated as: number of retrieved references by the patient preferences domain/(number of non-utility studies + qualitative studies)

^c^The precision for the utility values domain is calculated as: number of studies on utility measures/number of retrieved references by the utility values domain. The NNR is calculated as: number of retrieved references by utility values domain/number of studies on utility measures

**Table 7:** Use of the search strategy in the development of a healthcare guideline

Allergic rhinitis in asthma guideline^a^	Retrieved references (n)	Relevant references (n)	Relevant studies on non-utility measures	Relevant studies on utiltiy measures	Relevant qualitative research	Precision (%) 95% CI	NNR
Global search strategy	1560	33	27	6	0	2,12 (1.51-2.96)	47,27
Patient preferences domain	1487	31	27	4	0	1,82^b^ (1.25-2.63)	55,07^b^
Utility values domain	92	7	1	6	0	6,52c (3.02-13.51)	15,33^c^

^a^Search period: from inception until May 1st 2015

^b^The precision for the patient preferences domain is calculated as: (number of non-utility studies + qualitative studies)/number of retrieved references by patient preferences domain. The NNR is calculated as: number of retrieved references by patient preferences domain/(number of non-utility studies + qualitative studies)

^c^The precision the utility values domain is calculated as: number of studies on utility measures/number of retrieved references by utility values domain. The NNR is calculated as: number of retrieved references by utility values domain/number of studies on utility measures

**Table 8:** Use of the the search strategy in the update of a healthcare guideline

Care during pregnancy and puerperium guideline^a^	Retrieved references (n)	Relevant references (n)	Relevant studies on non-utility measures	Relevant studies on utility measures	Relevant Qualitative research	Precision (%) 95% CI	NNR
Global search strategy	668	19	9	1	9	2.84 (1.83-4.40)	35.2
Patient preferences domain	653	19	9	1	9	2.76^b^ (1.75-4.31)	36.3^b^
Utility values domain	22	2	2	0	0	0	NA

^a^Search period: First cycle from 01/01/2012- 31/08/2014; Second cycle from 01/09/2014 to 28/02/2015; Third cycle from 01/03/2015 to 31/08/2015

^b^The precision for the patient preferences domain is calculated as: (number of non-utility studies + qualitative studies)/number of retrieved references by patient preferences domain. The NNR is calculated as: number of retrieved references by patient preferences domain/(number of non-utility studies + qualitative studies)

The search used for the systematic review about the relative importance of outcomes in COPD patients showed a low precision (2%, resulting from 252 relevant references from the 12,574 retrieved) and a NNR of 50 references. When the search was used to conduct a review for the development of a guideline on allergic rhinitis the strategy showed a precision of 2.12% (33 relevant references out of 1560 retrieved) with a NNR of 47 references. The strategy used to complete a literature surveillance to update 123 recommendations on pregnancy care from a clinical guideline showed a similar precision 2.84% (19 relevant references out of 668 retrieved), with a NNR of 35 references. Finally the precision of the search for the development of a database on patients’ and other stakeholders’ views about health care was 5% (314 relevant references from 6231 retrieved) with a NNR of 20 references.

## Discussion

### Main findings

We have developed a content search strategy to systematically identify studies addressing patients’ views and preferences in Pubmed. The strategy includes terms to retrieve references about utilities (and relative disutilities), other quantitative measures of preferences, decisions distributions, and findings from qualitative studies.

We used the relative recall approach to test the performance of this strategy against a reference set of studies included in relevant systematic reviews. After an initial test of the strategy and the addition of some terms to the search string we obtained a relative recall of 85, 8%. We designed the search strategy to use two independent blocks of terms, one related to patients’ preferences and decision making, and one to utilities values. The former showed a better recall than the latter.

When conducting reviews the most relevant performance indicators for the search are relative recall (equivalent to sensitivity) and precision (equivalent to positive predictive value). We tested the precision of the search strategy in a series of knowledge synthesis projects. In all the cases the precision was low, ranging from 2% to 5%. These findings are in concordance with a standard practice in designing search strategies for synthesis of scientific evidence, where the optimization of recall comes at the price of a high reference screening burden [66, 67].

### Our results in the context of previous research

The concept of how patients value different health care issues is broad and complex, and can be interpreted from multiple approaches, with several research designs providing valuable information. This variability of conceptual frameworks to approach the topic and study methodologies makes it challenging to identify relevant studies. Furthermore, this area of knowledge is continuously evolving and the terminology used is still immature, adding further challenges to the searching process. Most studies use somewhat different terminology to refer to the same issue, and there is lack of systematic indexing for this topic [17].

The search strategy we present takes into consideration the complexity of conducting an evidence synthesis about this topic, and the need to use a broad vocabulary to ensure its comprehensiveness. We therefore incorporated search terms to retrieve the different measures available (e.g. utilities or qualitative findings), elicited directly and indirectly. It also includes terms to retrieve evidence on preferences, attitudes to health, patient decisions, participation, satisfaction, views or perceptions.

Other researchers have designed search strategies responding to the common necessity to identify studies on this topic but from different perspectives and scopes, making difficult an adequate comparison of their performance. The Scottish Intercollegiate Guidelines Network (SIGN) developed a search strategy for patient issues [27], containing over 200 terms that relate mostly to patients, carers, and relatives’ feelings, emotions, perceptions, concerns, and satisfaction, as well as evidence drawn from support, self-help, and social groups. To our knowledge this filter has not been validated and no details on how it was developed have been published [68, 69]. The Knowledge Institute of Medical Specialists (KiMS) developed a literature search filter specific for patients’ knowledge, views, and values [27]. This filter used the SIGN search as a starting point and was subsequently refined to focus on patient experiences, information needs, unfulfilled needs, preferences, participation in decision-making and satisfaction. The researchers assembled a gold standard to assess the sensitivity, specificity, and precision. Another recently published study [30], used a gold standard approach to validate a set of filters to retrieve studies focusing specifically in patients’ preferences for treatment outcomes, with an excellent performance. To our knowledge, the only filter that also used the relative recall approach to test its performance was restricted to the identification of studies that report on health state utilities [29]. The filter performance was similar to the obtained in our experience, with a slightly better recall (91%) and a lower precision (0.3%).

Our search strategy used in real life experiences obtained a low precision with an appreciable NNR. This is not surprising given the broad array of study designs, the methodologies that can be informative, and the broad vocabulary that has to be considered to improve the search comprehensiveness, which inherently increases the pool of references that may be eligible [17, 57]. This is consistent with the limitations of search strategies to retrieve studies in other fields such as qualitative research. A recent methodological review [70] identified four qualitative filters, developed for MEDLINE (using PubMed), EMBASE (Ovid), PsycINFO (Ovid), and CINAHL (Ovid). While the sensitivity and specificity of these filters were favourable, there are serious concerns with the comprehensiveness of the included terms [70, 71]. Similarly, a recent study [72] assessed the performance of search filters of qualitative research, including eight combinations for MEDLINE, seven for CINAHL, and four for Social Science Citation Index filters. Authors reported that overall precision was disappointingly low, and variable depending on the topic searched, either COPD or early breast cancer.

The design of a search strategy to retrieve studies with a low prevalence in the scientific literature, using an approach that aims to maximize sensitivity will invariably penalize its precision. Consistently, the precision derived from the use of our search is in the range of the precision showed by the searches in samples of systematic reviews [67].

### Limitations and strengths

The main strength of our study is that we adopted an explicit methodology to test the performance of our search strategy. We developed the search strategy in different steps, testing the relative recall and precision from independent sets of studies. First, we assembled terms in a comprehensive process, calculated the relative recall of the resulting search against a reference set of relevant studies that allowed us to improve the initial search string. We also tested the final strategy in different projects allowing us to value other important performance characteristics in the development of evidence syntheses.

It could be argued that the validity of relative recall is strongly conditioned by the capacity of the systematic reviews used as reference to adequately identify their included studies. We tried to mitigate this shortcoming retesting the search with a comprehensive set of relevant reviews, that provided at least 100 pertinent reference studies, against which the filter was compared [48, 49].

### Implications for practice and research

The availability of a standardised approach to retrieve studies on patients’ views and preferences will likely contribute to ease the use of this issue in the process of conducting systematic reviews, health technology assessments and clinical guidelines. This search strategy fills an important methodological gap and will enable the development of systematic reviews in this field.

In order to lower the burden associated with identifying this type of evidence researchers could implement the proposed search strategy by initially focusing on specific measures (e.g. utilities or qualitative findings) or study designs (e.g. standard gamble, time trade off, or visual analogue scales). Other less-burdensome strategies could entail focusing just on systematic reviews or on more recent or context-specific studies.

There are some valuable implications for future research after the development of this content search strategy. There is a need to validate our strategy with a gold standard approach to evaluate other performance characteristics such as specificity, and to obtain the terms with the best yield. Additionally, the adaptation and evaluation in other databases different from PubMed should also be conducted. Finally, it would be important to determine the relative performance of alternative strategies in this field.

## Conclusions

The proposed content search strategy designed for PubMed will help researchers to systematically identify relevant studies addressing patients’ views and preferences. This will facilitate the inclusion of this critical aspect when formulating heath care decisions, including recommendations.

## Additional file

Additional file 1: List of terms of the final version of the proposed search strategy. (DOCX 23 kb)

## Abbreviations

COPD: Chronic Obstructive Pulmonary Disease
