# Supplementary material for: Development and use of a content search strategy for retrieving studies on patients' views and preferences
Source: Health Qual Life Outcomes. 2017 Aug 30;15:126. doi: 10.1186/s12955-017-0698-5 (PMC5576198; doi:10.1186/s12955-017-0698-5)
Supplement: Additional file 1: — List of terms of the final version of the proposed search strategy. (DOCX 23 kb) [file 12955_2017_698_MOESM1_ESM.docx]

## Additional file 1. List of terms of the final version of the proposed search strategy

### Patient preferences and decision making domain

1. “Attitude to Health” [MAJR]

2. “Patient Participation”[MAJR]

3. “Patient Preference”[MAJR]

4. preference*[tiab]

5. choice[ti]

6. choices[ti]

7. value*[ti]

8. health state values[tiab]

9. valuation*[ti]

10. expectation*[tiab]

11. attitude*[tiab]

12. acceptab*[tiab]

13. knowledge[tiab]

14. point of view[tiab]

15. user participation[tiab]

16. users participation[tiab]

17. users' participation[tiab]

18. user's participation[tiab]

19. patient participation[tiab]

20. patients participation[tiab]

21. patients' participation[tiab]

22. patient's participation[tiab]

23. patient perspective*[tiab]

24. patients perspective*[tiab]

25. patients' perspective*[tiab]

26. patient's perspective*[tiab]

**27. user perspective*[tiab]**

**28. users perspective*[tiab]**

**29. users' perspective*[tiab]**

**30. user's perspective*[tiab]**

31. patient perce*[tiab]

32. patients perce*[tiab]

33. patients' perce*[tiab]

34. patient's perce*[tiab]

35. health perception*[tiab]

**36.user perce*[tiab]**

**37.users perce*[tiab]**

**38.users' perce*[tiab]**

**39.user's perce*[tiab]**

40. user view*[tiab]

41. users view*[tiab]

42. users' view*[tiab]

43. user's view*[tiab]

44. patient view*[tiab]

45. patients view*[tiab]

46. patients' view*[tiab]

47. patient's view*[tiab]

48. ((decision*[ti] AND mak*[ti]) OR decision mak*[tiab] OR decisions mak*[tiab]) AND (patient*[tiab] OR user*[tiab] OR men[tiab] OR women[tiab])

54 discrete choice*[tiab]

55. decision board*[tiab]

56. decision analy*[tiab]

57. decision-support[tiab]

58. decision tool*[tiab]

59. decision aid*[tiab]

60. discrete-choice*[tiab]

62. “Decision Making”[MAJR] AND (patient*[ti] OR user*[ti] OR men[ti] OR women[ti])

### Health state utility values domain

63. “Decision Support Techniques”[MeSH]

64. health[ti] AND utilit*[ti]

65. gamble*[tiab]

66. prospect theory[tiab]

67. preference score[tiab]

68 preference elicitation[tiab]

69. health utilit*[tiab]

70. utility value*[tiab]

71. utility score*[tiab]

72. Utility estimate*[tiab]

74. health state[tiab]

75. feeling thermometer*[tiab]

76. best-worst scaling[tiab]

77. standard gamble[tiab]

78. time trade-off[tiab]

79. TTO[tiab]

80. probability trade-off[tiab]

81. utility score[tiab]

82. preference based[tiab]

83. preference score*[tiab]

84. multiattribute[tiab]

85. multi attribute[tiab]

86. EuroQol 5D [tiab]

87. EuroQol5D[tiab]

88. EQ5D [tiab]

89. EQ 5D [tiab]

90. SF6D [tiab]

91. SF 6D [tiab]

92. HUI [tiab]

93. 15D[tiab]

### Additional terms added after the initial assessment.

knowledge[tiab]

user perspective*[tiab]

users perspective*[tiab]

users' perspective*[tiab]

user's perspective*[tiab]

user perce*[tiab]

users perce*[tiab]

users' perce*[tiab]

user's perce*[tiab]

.
